# Estimating the differential exposure of household groups to alcohol duty reforms in Great Britain

**DOI:** 10.1093/pubmed/fdaf081

**Published:** 2025-07-09

**Authors:** Luke B Wilson, Rob Pryce, Grace Leeming, John Holmes, Colin Angus

**Affiliations:** School of Medicine and Population Health, Regent Court, 30 Regent Street, University of Sheffield, Sheffield S1 4DA, UK; School of Medicine and Population Health, Regent Court, 30 Regent Street, University of Sheffield, Sheffield S1 4DA, UK; School of Medicine and Population Health, Regent Court, 30 Regent Street, University of Sheffield, Sheffield S1 4DA, UK; School of Medicine and Population Health, Regent Court, 30 Regent Street, University of Sheffield, Sheffield S1 4DA, UK; School of Medicine and Population Health, Regent Court, 30 Regent Street, University of Sheffield, Sheffield S1 4DA, UK

**Keywords:** alcohol, tax, exposure, alcohol duty reform, socioeconomic position

## Abstract

**Background:**

We explored the potential impact of changes to the UK alcohol tax system implemented in August 2023 on increases on consumer spending, the separate impacts of the changes to the duty structures, and how these impacts vary between households depending on their level of alcohol purchasing and their socioeconomic position.

**Methods:**

We used household-level purchasing data from Kantar’s Worldpanel to analyse four alternative scenarios, reflecting the three separate components of the duty reforms (the changes to the duty structures, the temporary wine easement, and the additional 10.1% increase in duty rates).

**Results:**

In the 12 months prior to the implementation of the reforms, the average household spend on off-trade alcohol in Kantar’s Worldpanel was £324.37 (August 2022–July 2023). This average conceals a heavily skewed distribution, with the lowest-purchasing 20% of households spending an average of £20.47 per year compared to £1206.68 for the highest-purchasing quintile. On average, households in higher socioeconomic position spend more on alcohol than those in lower socioeconomic positions—£339.19 compared to £302.37.

**Conclusion:**

Our results provide support for the structural reforms to alcohol duty introduced in the UK being effectively targeted at the heaviest alcohol purchasers, with no evidence to suggest that they are likely to increase economic inequalities.

## Introduction

Alcohol places a substantial burden on society, with the total social cost in England estimated at £27.4 billion each year.^1^ This cost comprises the negative impacts of alcohol on public health and associated costs of health and social care services as well as costs to the police and criminal justice system and the economic costs of lost productivity. The health burden of alcohol in particular has been rising in England over recent decades and has risen particularly sharply during the COVID-19 pandemic, with age-standardized mortality rates for alcohol-specific conditions rising by 34.3% between 2019 and 2022.^2^

Increasing alcohol prices is one of the most effective policy approaches available to decision makers seeking to reduce the burden of alcohol harms and are included in the World Health Organization’s ‘Best Buys’.^3^ Alcohol taxation is the primary mechanism through which governments can influence alcohol prices and most countries levy some form of duties on alcohol. Alcohol duty (or tax) is paid for by the company that produces or imports the products and is included in the price to the consumer. This generates revenue for the government. However, not all alcohol taxation systems are equal, with the effect of any system of alcohol duty depending on both the level at which duties are set as well as the structure of those duties, i.e. the basis on which products are taxed.

There are three main ways in which alcohol can be taxed: specific taxation where products are taxed on the basis of their alcohol content, unitary taxation where products are taxed on the basis of their volume, and ad valorem taxation, where products are taxed on the basis of the sales price. Most countries, including the UK (prior to August 2023), implement hybrid systems that combine specific, unitary, and ad valorem taxation.

Meta-analyses of over 100 time series and panel studies consistently find that increasing alcohol prices is associated with a reduction in alcohol sales.^4–7^ In addition, further meta-analyses have suggested that price increases reduce both acute and chronic alcohol harms.^8^ There is little evidence to date on the relative effectiveness of alcohol taxation systems, or their interaction with other pricing policies and are currently no studies that have evaluated the impact of reforms comparable to those implemented in the UK. However, model-based studies of hypothetical systems suggest that, all else being equal, taxing drinks in proportion to their alcohol-by-volume (ABV) can reduce alcohol harms and health inequalities across socioeconomic positions, but may raise less revenue than other approaches (e.g. taxing drinks in proportion to their price).^9–12^ However, there have been several studies that examine how alcohol tax changes affect prices paid by consumers. Studies from the UK, USA, Belgium, and Denmark find that alcohol prices increase by as much or more than implied by tax changes in the off-trade (i.e. shop-bought) and on-trade (e.g. pubs, restaurants, nightclubs).^13–17^

**Figure 1:**
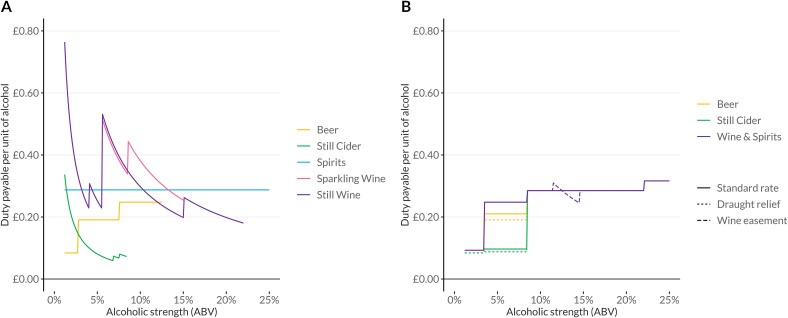
Duty rates payable per unit in the UK under the previous (panel a) and new (panel b) alcohol duty systems.

In Autumn 2021, following an evidence review, the UK Government announced a reform of its alcohol duty system, including proposals to tax all products on a specific basis.^18^ Further, under the new system, stronger products would be taxed at higher rates, reflecting evidence that higher ABV drinks are both disproportionately drunk by heavier drinkers^19^ and associated with greater levels of intoxication.^20^  Figure 1 shows the structures of the old and new duty systems payable per unit of alcohol. One UK unit of alcohol equals 10 mL or 8 g of pure alcohol. Note that all alcoholic products are also eligible for an ad valorem Value Added Tax (VAT) set at 20%. This is the UK’s general sales tax and applies to most goods and services. It is therefore not reflected in these figures.

Upon their announcement, these reforms aimed to simplify and rationalize the tax system and were explicitly linked to a desire to improve public health.^18^ However, in spite of following these principles recommended by the World Health Organization, the proposed new system did not fully align with public health guidance. Most notably the rates of duty for cider between 3.4% and 8.4% ABV remained substantially lower than other products at similar strengths—less than half of the rate for beer, for example. This is partly a result of long-term efforts to support cider producers in politically important areas in the South-West of England. The proposals also included a small reduction in the duty rate for ‘draught’ products—primarily beer—served from a cask or keg rather than from a bottle or can in the on-trade. This is known as ‘draught relief’. Finally, following intense lobbying by the wine industry, a temporary ‘easement’ was granted for wine to reduce the administrative burden associated with the introduction of the new system. Under this easement, all wine between 11.5% and 14.5% is taxed as if it were 12.5% ABV from August 2023 to February 2025, removing the explicit link between alcohol content and duty payable. See Supplementary Figure 1 to Supplementary Figure 5 for the expected changes in duty per unit by beverage type.

Prior to the implementation of the new duty structures in August 2023, the UK Government also announced that there would be a concurrent 10.1% increase in all duty rates.^21^ This increase reflected the very high levels of general price inflation globally in the preceding 12 months, referred to in the UK as the ‘cost-of-living crisis’.

Although these duty reforms and increases have now been implemented, there has been little research to date to understand their potential impact on consumer spending, and none on the extent to which different groups in society were exposed to any resulting price increases. This paper aims to address this gap by estimating: (i) the potential impact of the UK duty reforms and increases on consumer spending; (ii) the separate impacts of the changes to the duty structures, changes to duty levels, and the wine easement, and (iii) how these impacts vary between households depending on their level of alcohol purchasing and their socioeconomic position. For data availability reasons, it focuses only on the off-trade alcohol and therefore does not consider draught relief. However, data from 2022 shows that off-trade sales account for 73% of all alcohol sales in the UK by volume.^22^

## Methods

### Data

This analysis used data collected by the market research company Kantar through their Worldpanel platform (henceforth KWP). KWP is a rolling panel of ~30 000 households across Great Britain (England, Scotland, and Wales), designed to be representative of the general population. The KWP sample is recruited through stratified quota sampling, with quotas set for geographical region, household size, age of main shopper, and occupational social grade. The same households provide longitudinal data over time, with continuous replenishment to replace households that leave the sample and ensure the panel remains representative of households in Great Britain. Participating households that drop out of the panel are replaced, with a mean participation length of 18 months. Participating households record the price and full product details all food and drink products brought into the home, including the volume purchased, product type, and brand. Kantar also collects household-level data including household composition, household income, and the primary shopper’s occupation.

We analysed data on alcohol purchases from August 2022 to July 2023, the 12 months immediately prior to the implementation of the duty reforms, in order to capture seasonal variations in both prices and purchasing patterns. This gave us an analytical sample of 12 866 households, of whom 89.1% (*N* = 11 464) bought alcohol at some point during the year. For each household, we retained all purchases involving alcoholic products with an ABV of at least 1.2% as products with an ABV <1.2% are not eligible for duty. All prices were inflated to August 2023 levels using monthly Consumer Price Index including owner occupiers' housing costs (CPIH) inflation figures.^23^

### Analysis

We analysed four alternative scenarios, reflecting the three separate components of the duty reforms (the changes to the duty structures, the temporary wine easement, and the additional 10.1% increase in duty rates):

(1) Duty structure changes only, with wine easement
(2) Duty structure changes only, without wine easement
(3) Duty structure and rate changes, with wine easement
(4) Duty structure and rate changes, without wine easement

Of these, Scenario 1 represents the revised proposal following consultation in September 2022.^24^ Scenario 2 represents the original proposal announced in the Autumn 2021 Budget.^18^ Scenario 3 reflects the reforms as implemented in August 2023 and Scenario 4 represents the proposed system from February 2025 onwards.

In order to analyse the exposure of households in KWP to price increases under each of the scenarios, we began by calculating the amount of duty payable on every purchase in the dataset prior to the intervention. We did this using information on the product type (beer, still wine, sparkling wine, cider, or spirits) and ABV, using the alcohol duty rates in place in July 2023.^25^ This calculation also included the VAT payable on the duty component only to obtain the total amount of duty payable on each purchase. We then calculated the duty that would be payable in each of our four scenarios based on the relevant duty structure and rates post-intervention.

The purchases for each household were then combined to derive each household’s total annual spend on alcohol in the 12 months prior to the duty reforms and the exposure (i.e. the amount of extra spending) implied by each of the reform scenarios. We report the absolute and relative exposure for each scenario for the overall population and for the following population subgroups: (i) alcohol purchasing quintiles derived from the average number of units of alcohol purchased per adult household member, excluding households who did not purchase any alcohol; (ii) socioeconomic position classified using social grade. Social grade is a way of grouping people mainly based on their social and financial situation (https://www.ons.gov.uk/census/aboutcensus/censusproducts/approximatedsocialgradedata). We dichotomized this as high—households whose main alcohol purchaser had an occupation categorized as managerial, administrative, or professional, and low—where their occupation was categorized as manual or other, or who were unemployed and (iii) households in poverty and those not in poverty, on the basis of households whose equalized income falls below 60% of the median for the population.^26^ Due to the relatively large number of households that did not report income data, we also include an ‘unknown’ poverty category.

## Results

### Pre-intervention spending on alcohol

In the 12 months prior to the implementation of the duty reforms, the mean household spend on off-trade alcohol was £324.37 from August 2022 to July 2023 in KWP. This average conceals a heavily skewed distribution, with the lowest-purchasing 20% of households spending an average of £20.47 per year compared to £1206.68 for the highest-purchasing quintile. Table 1 presents a full breakdown of the pre-intervention spending patterns. On average, households in higher socioeconomic positions spend slightly more on alcohol than those in lower socioeconomic positions—£339.19 compared to £302.37, with a slightly larger gap between households that are and are not in poverty—£333.30 compared to £273.99.

**Table 1:** Household spending on off-trade alcohol in the 12 months prior to the duty reforms.

Population group		Number of households purchasing alcohol	Mean annual spend on alcohol pre-reform	Mean annual number of units of alcohol purchased pre-reform
Population		11 402	£324.37	357.8
Purchasing quintile	Lowest	2281	£20.47	24.4
	Lower	2280	£66.12	92.4
	Middle	2281	£163.33	246.2
	Higher	2280	£376.27	618.5
	Highest	2280	£1206.68	2272.6
Socioeconomic position	Higher (ABC1)	6920	£339.19	589.5
	Lower (C2DE)	4482	£302.37	555.4
Household poverty	Not in poverty	7669	£333.30	586.8
	In poverty	1829	£273.99	513.1
	Unknown	1904	£340.18	595.9

### Household exposure to impacts of the intervention on spending on alcohol

The exposure to increases in spending under each of the modelled duty reform scenarios are shown in Table 2. At a population level, the changes to the duty structure alone (Scenario 2) are estimated to increase the average annual spend for households that buy alcohol by £1.91, a 0.59% increase. Adding in the effect of the wine easement (Scenario 2) reduces this increase to £0.87 (+0.27% relative to pre-intervention). The impact of the increase in duty rates (Scenario 3) is substantially larger, increasing household spending by an average of £17.42 per year, a 5.37% rise, and this is estimated to increase further to a total increase of £18.58 (+5.73%) when the wine easement is removed.

**Table 2:** Changes in annual household spending under each modelled duty reform scenario.

Population group		Change in annual spend on alcohol (%)			
		Scenario 1	Scenario 2	Scenario 3	Scenario 4
Population		+£0.87 (0.27%)	+£1.91 (0.59%)	+£17.42 (5.37%)	+£18.58 (5.73%)
Purchasing quintile	Lowest	−£0.39 (−1.90%)	−£0.37 (−1.81%)	+£0.26 (1.25%)	+£0.28 (2.51%)
	Lower	−£0.98 (−1.48%)	−£0.88 (−1.32%)	+£1.55 (2.35%)	+£1.66 (2.51%)
	Middle	−£1.85 (−1.13%)	−£1.57 (−0.96%)	+£4.97 (3.05%)	+£5.29 (3.24%)
	Higher	−£0.89 (−0.24%)	+£0.31 (0.08%)	+£16.58 (4.41%)	+£17.90 (4.76%)
	Highest	+£9.0 (0.75%)	+£13.32 (1.10%)	+£75.10 (6.22%)	+£79.86 (6.62%)
Socioeconomic position	Higher (ABC1)	+£1.17 (0.34%)	+£2.46 (0.72%)	+£18.25 (5.38%)	+£19.67 (5.80%)
	Lower (C2DE)	+£0.42 (0.14%)	+£1.11 (0.37%)	+£16.19 (5.36%)	+£16.95 (5.61%)
Household poverty	Not in poverty	+£0.91 (0.27%)	+£2.02 (0.61%)	+£17.80 (5.34%)	+£19.02 (5.71%)
	In poverty	+£0.50 (0.18%)	+£1.15 (0.42%)	+£14.93 (5.45%)	+£15.65 (5.71%)
	Unknown	+£1.07 (0.31%)	+£2.28 (0.67%)	+£18.47 (5.43%)	+£19.80 (5.82%)

**Figure 2:**
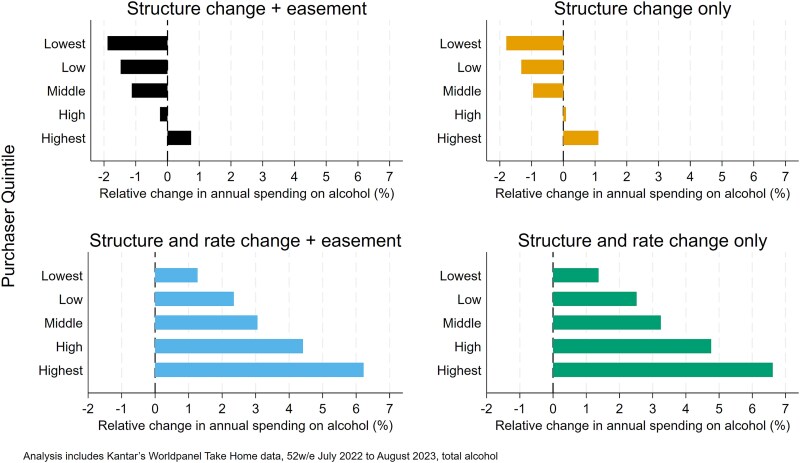
Relative impacts of duty reform scenarios on household spending on alcohol by purchaser quintile.

When we break these changes down by purchasing quintile, as illustrated in Fig. 2 (relative impact), it becomes clear how much exposure to price increases arising from the duty reforms is skewed towards households that buy the most alcohol. In absolute terms this is unsurprising, since households that buy the most alcohol would expect to see the biggest increases in spending through any increase in price. However, the fact that this pattern persists in the relative impacts highlights that the reforms are also increasing the prices of the products that are purchased disproportionately by households that buy more alcohol. In contrast, the structural reforms to duty lead to an overall reduction in spending on alcohol for all but the heaviest purchasing 20% of households. The marginal impact of adding in the wine easement is to slightly reduce the increase in spending among the heaviest purchasing households.

**Figure 3:**
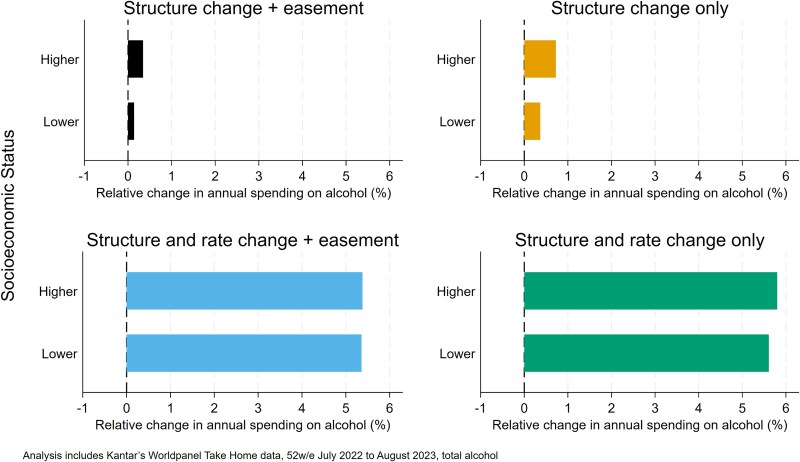
Relative impacts of duty reform scenarios on household spending on alcohol by socioeconomic position.

The extent to which exposure to the duty reforms varies across socioeconomic positions is illustrated in Fig. 3. This demonstrates that households with higher socioeconomic positions are more exposed to the reforms—facing a larger impact from the structural changes alone (+0.34% compared to +0.14% under Scenario 1) although this gap is narrowed once the impact of the increase in duty rates is accounted for (+5.38% compared to +5.36% under Scenario 3).

**Figure 4:**
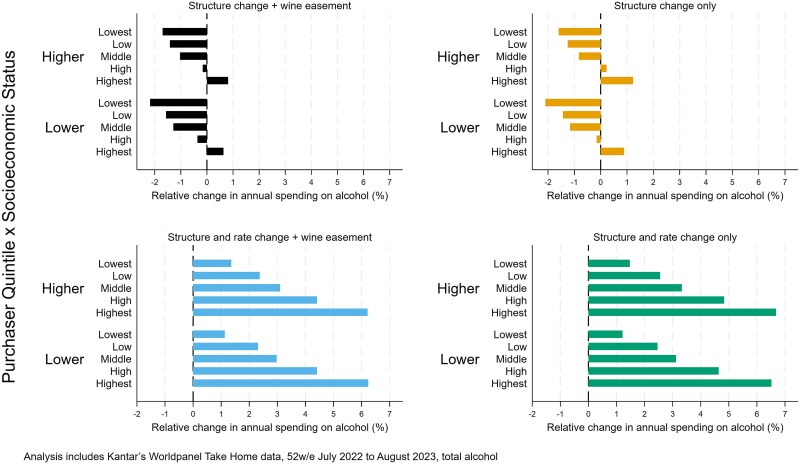
Relative impacts of duty reform scenarios on household spending on alcohol by socioeconomic position and purchaser quintile.

Stratifying our results by both socioeconomic position and purchaser quintile is illustrated in Fig. 4 (see Supplementary Table S1 for full results) shows that variation between purchasing quintiles is far greater than variation between socioeconomic positions and suggests that at least some of the socioeconomic position differences may be driven by how the purchasing quintiles are distributed across each socioeconomic position. Results for household poverty rather than socioeconomic position are very similar (see Supplementary Table S1 and Supplementary Figs S6 and S7).

## Discussion

### Main finding of the study

This study examined the absolute and relative changes in consumer spending implied by the alcohol duty reforms enacted in the UK in August 2023 across different groups in society. It examines four scenarios related to the reforms, isolating the impact of the structural change to the duty system, the increase in duty rates due to inflation, as well as the introduction of wine easement introduced after lobbying by the wine industry. Given that the stated aim of these reforms were to improve public health, understanding the exposure of different population groups to the reforms is important to assess the potential for this goal to be achieved. Our analysis shows that the structural reforms to the alcohol duty system—moving from a hybrid taxation approach to a fully specific one (Scenario 2)—are estimated to lead to a small increase in average household spending on alcohol (0.59%), but this is concentrated in the heaviest 20% of purchasing households, with spending estimated to decline in the remaining 80% of households. This suggests that the reforms are effectively targeted at those purchasing the most alcohol without ‘penalizing’ those drinking at lower levels, although this distributional effect is moderated slightly by the inclusion of the wine easement in the reforms. The effect of the structural reforms is, however, dwarfed by the effect of the increases in duty rates. After accounting for these rate increases, the estimated effect of the policies introduced in August 2023 shifts to a 5.37% rise in household spending on alcohol, although the largest impacts remain in the heaviest purchasing groups in both relative and absolute terms. The structural reforms are estimated to have the greatest impacts on higher socioeconomic positions, although this is less clearly the case once the duty rate increases are factored into the picture.

### What is already known on this topic

This study examines the implied effects of the UK duty reforms. To date, only one study has looked at the observed impact. This found that the reforms were associated with reductions in ABV for some beers, and an overall increase in alcohol prices.^27^ A key strength of our analysis is therefore its use of detailed panel data from a large sample to understand the exposure of different household groups to these changes. More broadly, although there is extensive evidence on the impacts of changes in *levels* of alcohol taxation, changes in the *structures* of alcohol duty systems are much rarer and this is one of the first studies to examine the impact of implemented reforms.

### Limitations of the study

There are also some limitations to note. KWP is longitudinal purchasing diary and respondents are only asked to record purchases that they bring back into the home. Therefore, we have no information on alcoholic beverages purchased out of home in the on-trade. However, data from 2022 show that off-trade sales account for 73% of all alcohol sales in the UK by volume.^22^ KWP data are household rather than individual-level, which allows us to fully understand the potential impact of the reforms on household finances. However, this means that we cannot assess the impact on specific individual groups in the population (e.g. by age or sex). We also only have data on alcohol purchasing, rather than consumption, although at the household level these are likely to be very strongly correlated. Another limitation is that the duty reforms may incentivize producers to reduce the strength of their products in response to the tax rises. The aim of this paper is to understand what role the reforms had on the potential impact on consumer spending and the extent to which different groups in society were exposed to any resulting price increases. Reductions in alcoholic strength of beverages are another, non-financial, way consumers might be exposed to this intervention and this would need examining in future research paired alongside market research data. Finally, our analysis assesses the *exposure* of each household to the duty reforms. In reality, previous econometric evidence suggests many consumers will respond to the reforms by changing their purchasing behaviour—switching to cheaper products, or reducing their purchasing.^4^ It is likely that the nature and extent of these differences will also vary across the population. We aim to explore these differences in future work which will use KWP data from the 12 months after the reforms were implemented to see how purchasing behaviour has changed at the individual household level. Our study focuses on the 12 months prior to the implementation of the duty reforms. However, prior to the reforms, the government ran a consultation on the new alcohol duty system from October 2021 which outlined the proposals for the duty reforms.^28^ This allowed producers the opportunity to adjust their products through reformulation prior to the reforms implementation in August 2023. However, evidence on reformulation has shown that the mean price per 10 ml of alcohol and per litre of product was significantly higher after the new tax system was implemented for beer, cider, and spirits and significantly lower for RTDs meaning that producers waited until the reforms occurred to implement change.^27^

### What this study adds

Our results provide some support for the structural reforms to alcohol duty introduced in the UK in August 2023 being effectively targeted at the heaviest alcohol purchasers, with no evidence to suggest that they are likely to increase economic inequalities. The marginal impact of the wine easement is small, but withdrawing it as planned in February 2025 is likely to slightly increase the specific target of the reforms as the resulting price increases will fall primarily on those purchasing more alcohol and higher socioeconomic positions. The increase in alcohol duty rates is likely to have had a substantial impact on household spending on alcohol; however, it is important to place this increase in the wider context, with alcohol duty rates in the UK having been cut in cash or real terms almost every year for the past decade. Therefore, while our analysis suggests the potential for positive public health impacts of the duty reforms due to their targeted design, it is unlikely that the reforms alone will counteract the negative effects of the overall real-terms decline or have a significant impact on rising rates of alcohol harms.

## Supplementary Material

Supplementary_Materials_fdaf081

## Data Availability

Use of the Kantar’s WorldPanel data is allowed under the terms of the contract and nondisclosure agreement between Kantar and the University of Sheffield, which requires research outputs to be submitted to the data provider ahead of publication. The data providers’ right to request changes is limited to matters of accuracy regarding the data. The data provider played no further role in the research process, including in conception, design, analysis, interpretation, write-up or the decision to publish. Data will not be shared. Data used in these analyses are a commercial product licensed for use by the University of Sheffield and cannot be shared. The data are available for in-person inspection at the University of Sheffield by researchers on request. Analytical code can be shared on request.

## Notes

**Luke B. Wilson,** Dr

**Rob Pryce,** Dr

**Grace Leeming,** Ms

**John Holmes,** Professor

**Colin Angus,** Professor
