# Supplementary material for: Estimating the differential exposure of household groups to alcohol duty reforms in Great Britain
Source: J Public Health (Oxf). 2025 Jul 9;47(4):756–64. doi: 10.1093/pubmed/fdaf081 (PMC12669999; doi:10.1093/pubmed/fdaf081)
Supplement: Supplementary_Materials_fdaf081 [file supplementary_materials_fdaf081.docx]

**Supplementary Materials**

*S1: Intervention impacts on alcohol prices*

The product-level variation in how the duty reforms affect the tax payable on alcohol is illustrated in Figure 2, broken down by product type: beer, cider, wine, spirits and RTDs (Ready-To-Drinks – pre-mixed spirit or wine-based drinks often called ‘alcopops’). Each bubble represents a group of products which face the same absolute change in price, with the bubbles sized in proportion to the total sales volume of the products affected. Panel A in the top-left represents Scenario 1. This demonstrates that the vast majority of beer products were unaffected by the reforms to duty structures, with only a small number of products sold above 8.5% ABV seeing a rise in duty and an even smaller number of products sold below 3.5% ABV or between 7.5% ABV and 8.5% ABV seeing a duty cut. Lower strength ciders below 4.5% ABV saw a small duty cut, with higher strength ciders facing a small rise. For wine the picture is more complicated, with the easement policy meaning wines below 11.5% ABV see a duty cut and wines above 11.5% ABV see a small increase. Note that although duty is levied on the specific ABV of the product, there is a 0.5% permissible discrepancy between the stated and actual strength, so it is typical for wines to be labelled in 0.5% ABV increments (11%, 11.5%, 12% etc.). For spirits, products below 22% ABV, which are typically liqueurs, saw a small duty cut, with no change for stronger products RTDs, which are typically around 5% ABV saw a larger reduction in duty. This is particularly the case for spirits-based products as they were previously taxed at the same rate as 40% ABV spirits.

Figure S1: Changes in alcohol prices for all four Scenarios. Bubbles are sized according to the number of products whose price changes by that value


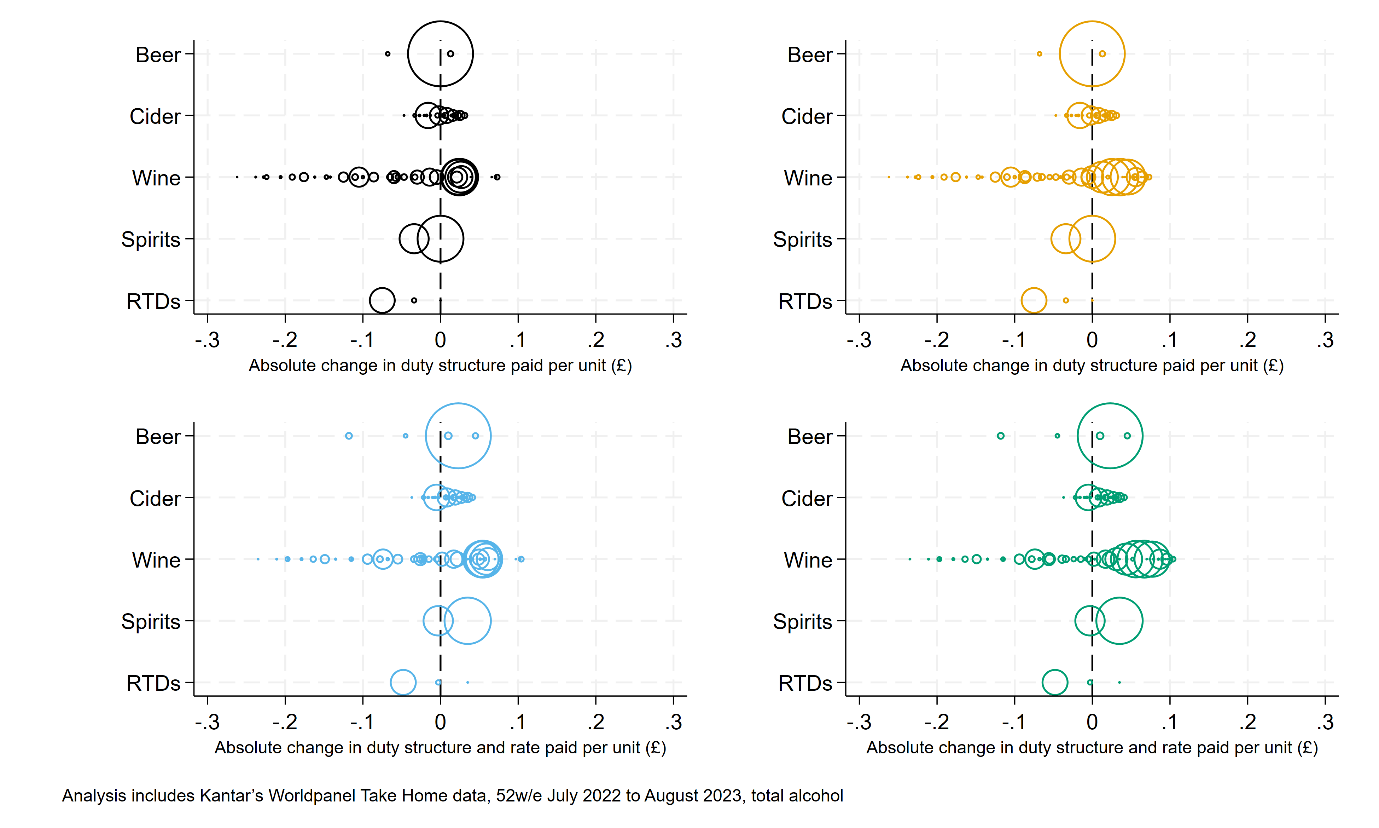


*Panel A (Top-left) Scenario 1, Panel B (Top-right) Scenario 2, Panel C (Bottom-left) Scenario 3, and Panel D (Bottom-right) Scenario 4.*

Figure S2: Changes in alcohol prices under Scenario 1. Bubbles are sized according to the number of products whose price changes by that value


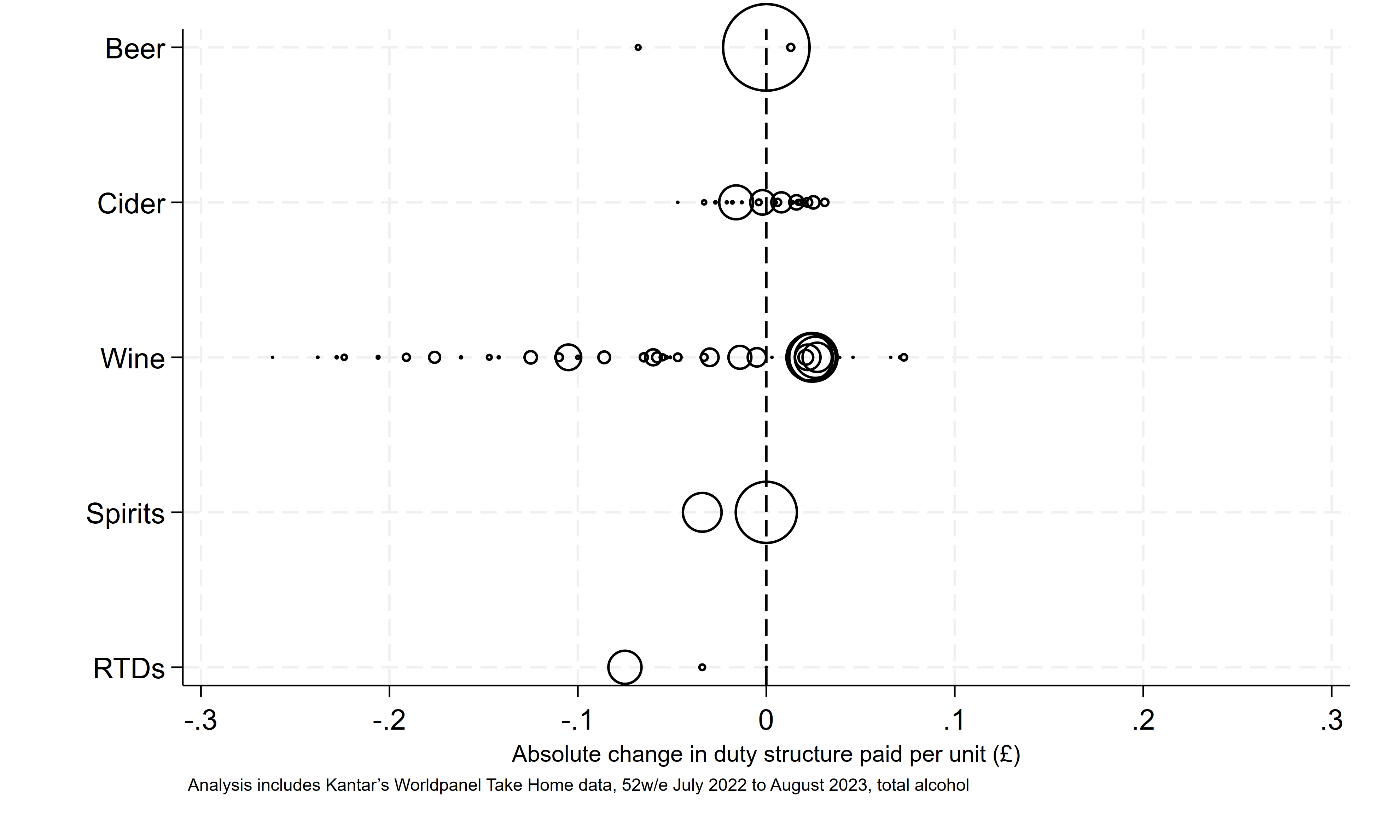


Figure S3: Changes in alcohol prices under Scenario 2. Bubbles are sized according to the number of products whose price changes by that value


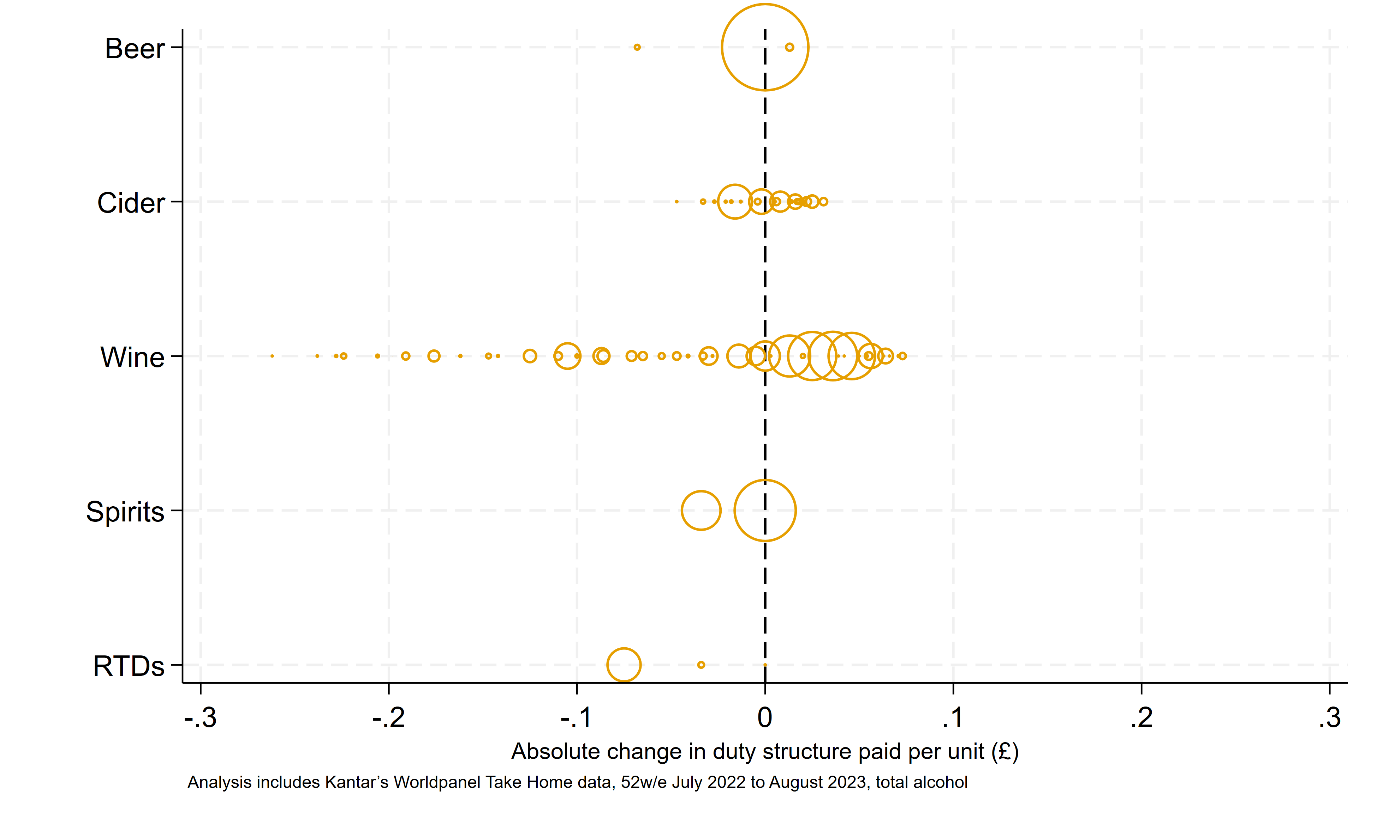


Figure S4: Changes in alcohol prices under Scenario 3. Bubbles are sized according to the number of products whose price changes by that value


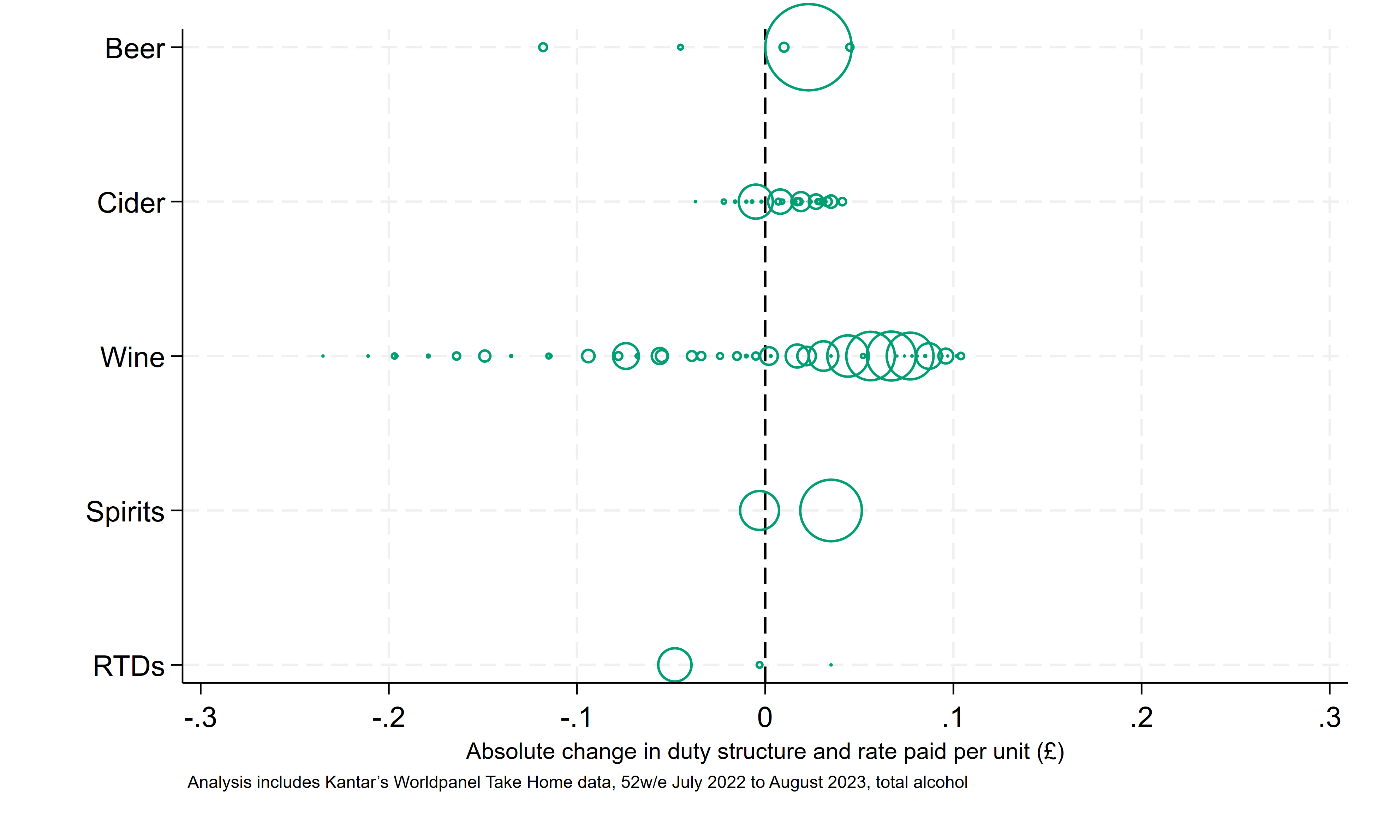


Figure S5: Changes in alcohol prices under Scenario 4. Bubbles are sized according to the number of products whose price changes by that value


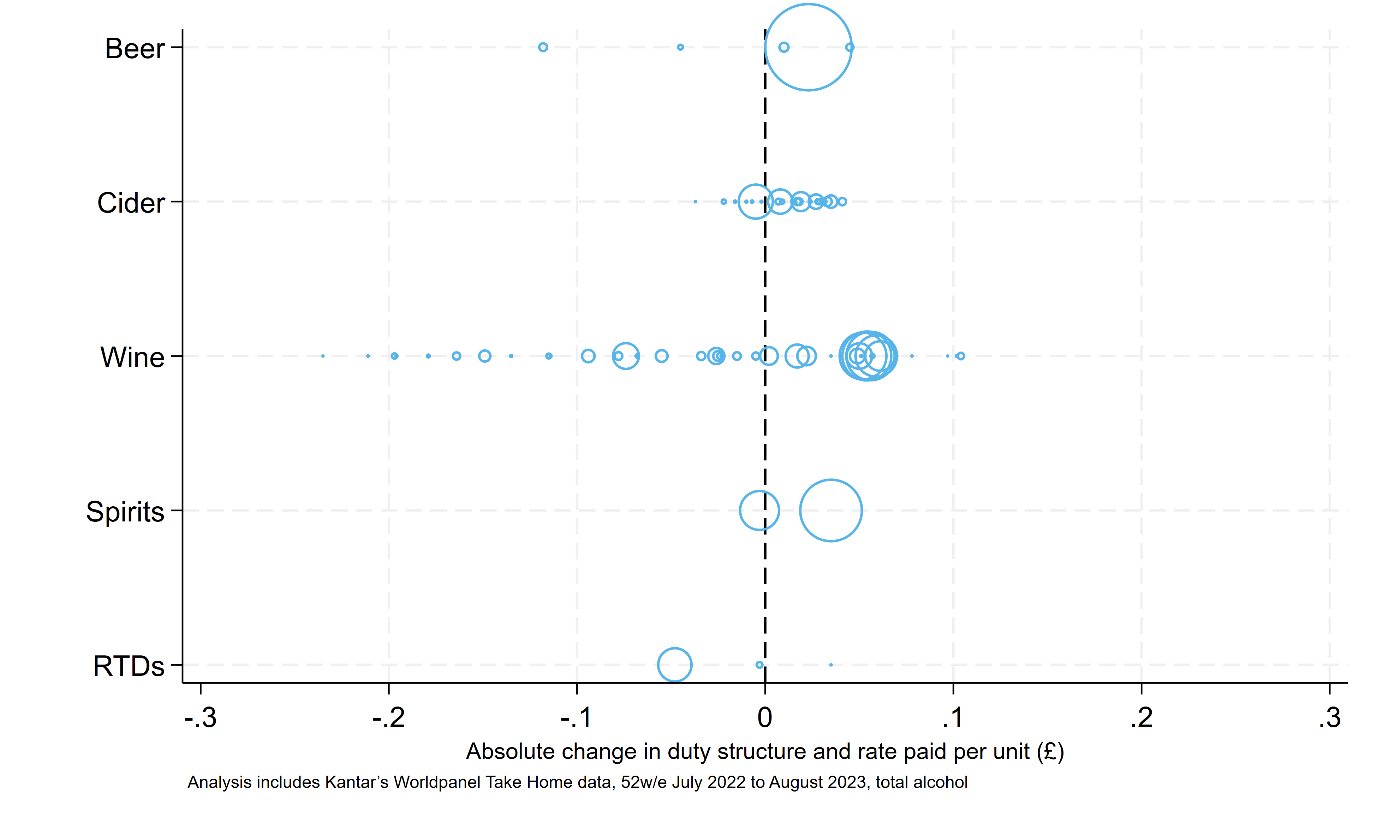


Table S1: Changes in annual household spending by socioeconomic/income position and purchaser quintile under each modelled duty reform scenario

| **Population group** | | **Change in annual spend on alcohol (%)** | | | |
| --- | --- | --- | --- | --- | --- |
| **Socioeconomic position** | **Purchaser quintile** | **Scenario 1** | **Scenario 2** | **Scenario 3** | **Scenario 4** |
| Higher (ABC1) | Lowest | -0.36 (-1.73%) | -0.34 (-1.62%) | +0.29 (1.38%) | +0.31 (1.49%) |
|  | Lower | -0.94 (-1.41%) | -0.83 (-1.25%) | +1.57 (2.37%) | +1.69 (2.55%) |
|  | Middle | -1.74 (-1.04%) | -1.4 (-0.84%) | +5.16 (3.09%) | +5.54 (3.31%) |
|  | Higher | -0.59 (-0.16%) | +0.84 (0.22%) | +16.68 (4.41%) | +18.25 (4.83%) |
|  | Highest | +9.82 (0.81%) | +14.93 (1.23%) | +75.23 (6.21%) | +80.85 (6.68%) |
| Lower (C2DE) | Lowest | -0.43 (-2.14%) | -0.41 (-2.07%) | +0.22 (1.08%) | +0.23 (1.16%) |
|  | Lower | -1.04 (-1.57%) | -0.95 (-1.44%) | +1.52 (2.3%) | +1.62 (2.45%) |
|  | Middle | -2.02 (-1.29%) | -1.83 (-1.16%) | +4.68 (2.98%) | +4.9 (3.12%) |
|  | Higher | -1.38 (-0.37%) | -0.57 (-0.15%) | +16.41 (4.4%) | +17.31 (4.64%) |
|  | Highest | +7.63 (0.64%) | +10.65 (0.89%) | +74.89 (6.24%) | +78.21 (6.52%) |
| **Household poverty** | **Purchaser quintile** |  |  |  |  |
| Not in poverty | Lowest | -0.38 (-1.88%) | -0.36 (-1.77%) | +0.25 (1.25%) | +0.27 (1.36%) |
|  | Lower | -0.92 (-1.42%) | -0.81 (-1.25%) | +1.53 (2.36%) | +1.65 (2.55%) |
|  | Middle | -1.82 (-1.13%) | -1.5 (-0.93%) | +4.83 (2.99%) | +5.18 (3.21%) |
|  | Higher | -0.77 (-0.21%) | +0.46 (0.12%) | +16.27 (4.36%) | +17.62 (4.72%) |
|  | Highest | +8.75 (0.73%) | +13.12 (1.1%) | +74.01 (6.18%) | +78.82 (6.58%) |
| In poverty | Lowest | -0.42 (-2.28%) | -0.4 (-2.21%) | +0.23 (1.25%) | +0.24 (1.33%) |
|  | Lower | -1.04 (-1.54%) | -0.96 (-1.42%) | +1.63 (2.41%) | +1.72 (2.54%) |
|  | Middle | -1.78 (-1.14%) | -1.58 (-1.01%) | +5.06 (3.24%) | +5.29 (3.38%) |
|  | Higher | -0.3 (-0.08%) | +0.85 (0.24%) | +17.42 (4.87%) | +18.68 (5.23%) |
|  | Highest | +7.15 (0.59%) | +10.08 (0.84%) | +74.66 (6.19%) | +77.89 (6.45%) |
| Unknown | Lowest | -0.4 (-1.62%) | -0.38 (-1.55%) | +0.31 (1.28%) | +0.33 (1.36%) |
|  | Lower | -1.14 (-1.63%) | -1.05 (-1.5%) | +1.54 (2.2%) | +1.65 (2.35%) |
|  | Middle | -2.02 (-1.14%) | -1.81 (-1.02%) | +5.48 (3.09%) | +5.72 (3.23%) |
|  | Higher | -1.8 (-0.45%) | -0.67 (-0.17%) | +17.18 (4.28%) | +18.42 (4.59%) |
|  | Highest | +11.69 (0.94%) | +17.1 (1.37%) | +80.09 (6.43%) | +86.05 (6.91%) |
| Notes: Analysis includes Kantar’s Worldpanel Take Home data, August 2022-July 2023, total alcohol | | | | | |

Figure S6: Relative impacts of duty reform scenarios on household spending on alcohol by household poverty status


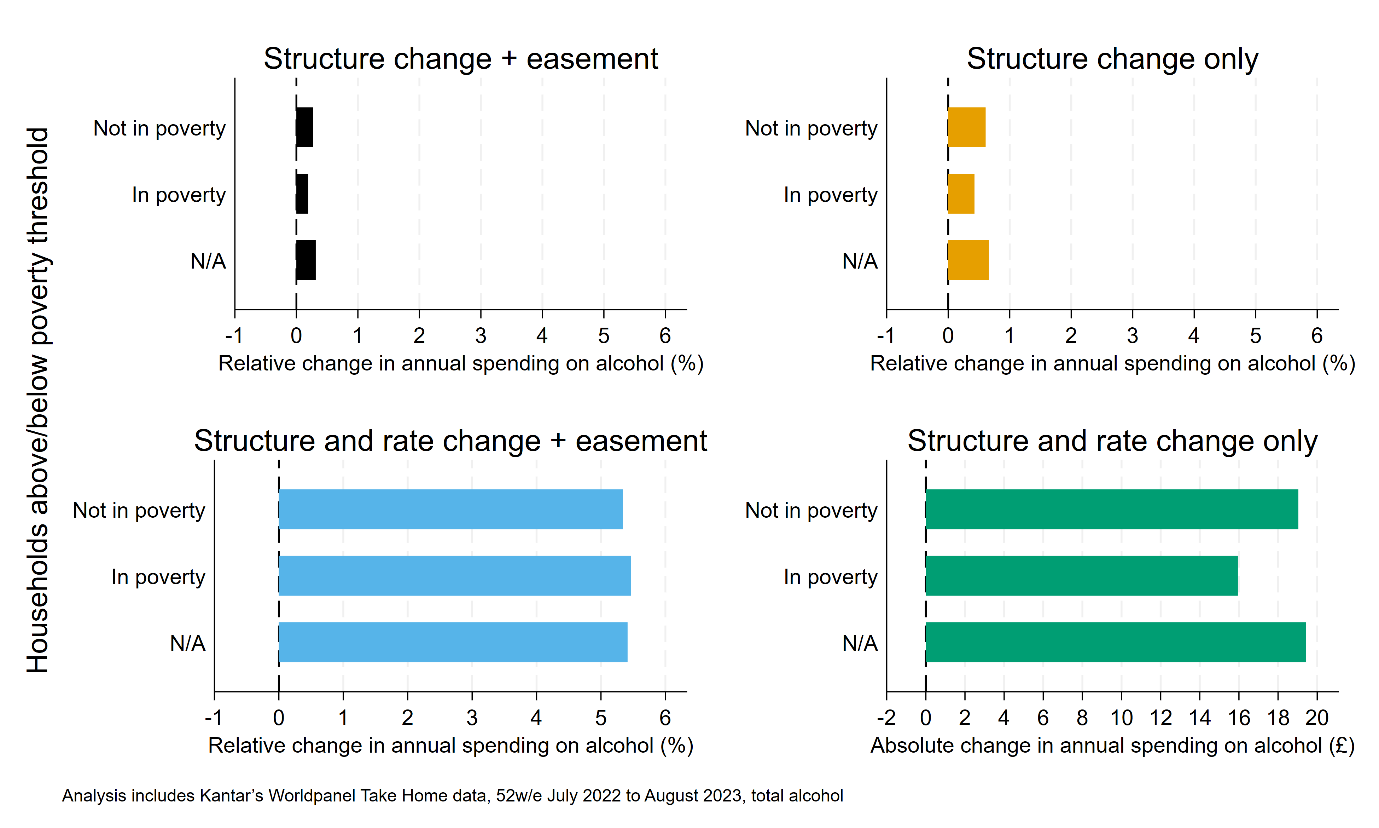


Figure S7: Relative impacts of duty reform scenarios on household spending on alcohol by household poverty status and purchaser quintile


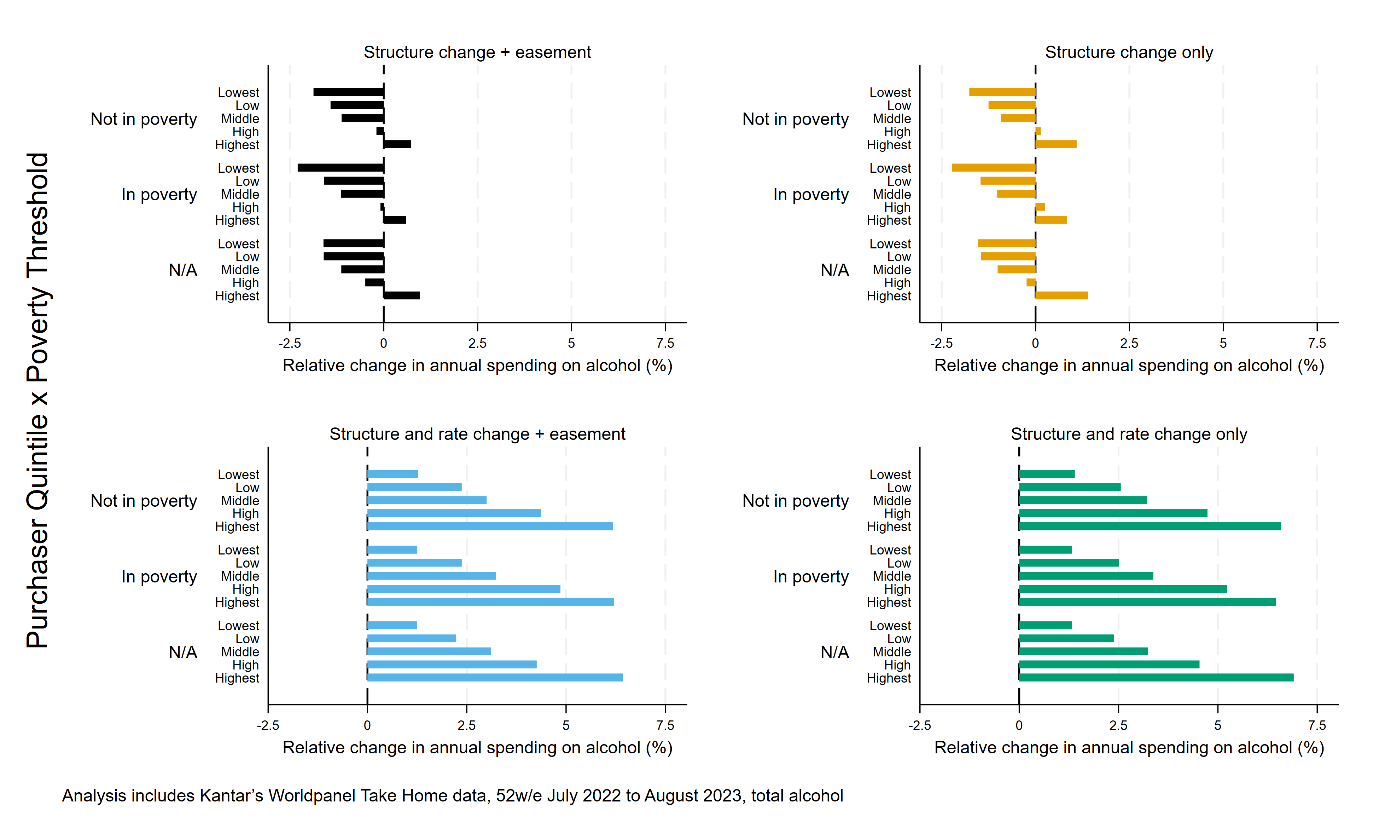


| *Table S2: Interactions between social class and poverty* | | | | |  |
| --- | --- | --- | --- | --- | --- |
| **Social Class** | **Poverty** | | | **Total** | **% Missing** |
|  | *Not in poverty* | *In poverty* | *N/A* |  |  |
| AB | 2,064 | 49 | 405 | 2,518 | 16.08% |
| C1 | 4,022 | 359 | 803 | 5,184 | 15.49% |
| C2 | 1,474 | 448 | 408 | 2,330 | 17.51% |
| D | 1,035 | 518 | 321 | 1,874 | 17.13% |
| E | 339 | 456 | 185 | 980 | 18.88% |
| **Total** | 8,584 | 2,180 | 2,122 | 12,886 | 16.47% |
| Notes: Analysis includes Kantar’s Worldpanel Take Home data, August 2022-July 2023, total alcohol | | | | | |
